# Supplementary figures and images for: Lipid-laden endothelial cells exhibit a transcriptomic signature linked to blood-brain barrier dysfunction, metabolic reprogramming and increased inflammation in the aging brain
Source: bioRxiv. 2025 Aug 28:2025.08.22.671845. Preprint. [Version 1] doi: 10.1101/2025.08.22.671845 (PMC12407747; doi:10.1101/2025.08.22.671845)

## Slide 1
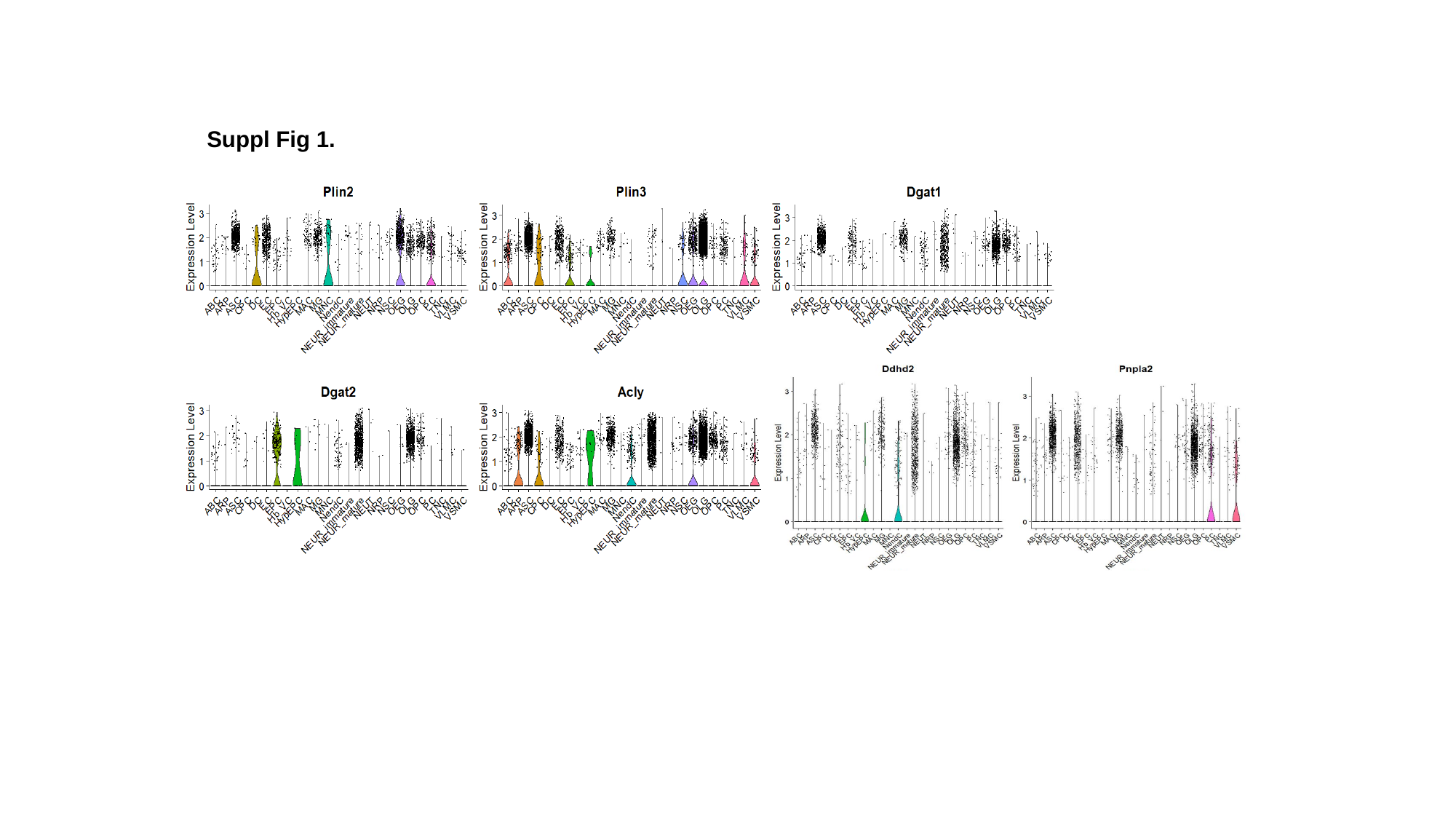

Suppl Fig 1.

Supplement: Supplement 1 — Suppl. Fig. 1: Violin plots showing expression of lipid metabolism-related genes in various brain cell types. [file media-1.pptx]
