# Supplementary material for: Lipid-laden endothelial cells exhibit a transcriptomic signature linked to blood-brain barrier dysfunction, metabolic reprogramming and increased inflammation in the aging brain
Source: bioRxiv. 2025 Aug 28:2025.08.22.671845. Preprint. [Version 1] doi: 10.1101/2025.08.22.671845 (PMC12407747; doi:10.1101/2025.08.22.671845)
Supplement: Supplement 2 — Suppl. Fig. 2: Lipid accumulation-induced changes in senescence and inflammation-related genes in ECs during aging. A-C) Density plots depicting the gene set enrichment scores for core senescence genes, SASP- and neuroinflammation-related genes, respectively, in Plin2-positive and negative brain ECs from the aged brains. [file media-2.pptx]

## Slide 1
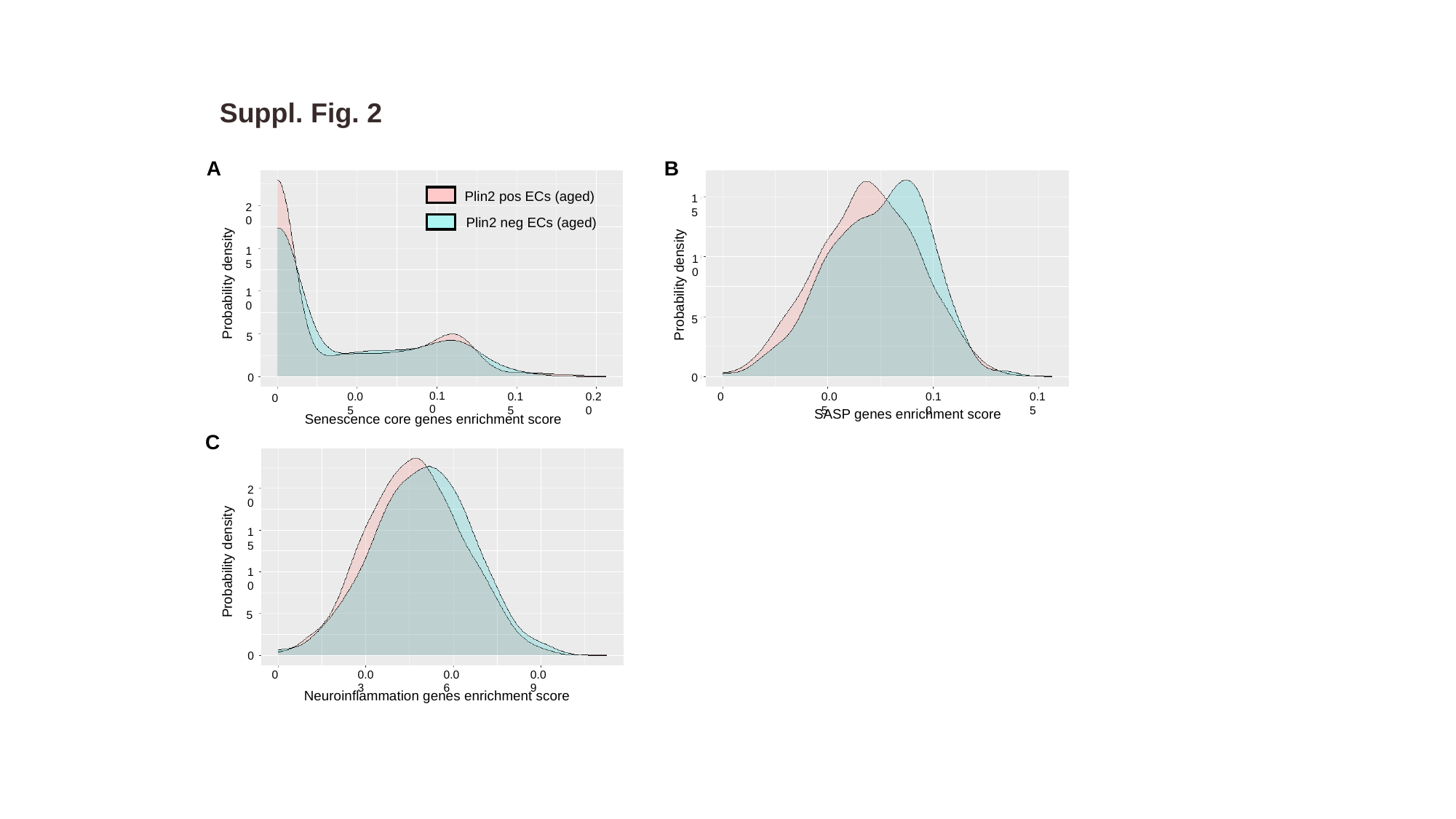

Suppl. Fig. 2
B
A
20
15
Probability density
10
5
0
0.10
0.05
0.15
0.20
0
Senescence core genes enrichment score
15
10
Probability density
5
0
0.10
0.15
0.05
0
SASP genes enrichment score
Plin2 pos ECs (aged)
Plin2 neg ECs (aged)
C
20
15
Probability density
10
5
0
0.03
0.06
0.09
0
Neuroinflammation genes enrichment score
