# Supplementary material for: Lipid-laden endothelial cells exhibit a transcriptomic signature linked to blood-brain barrier dysfunction, metabolic reprogramming and increased inflammation in the aging brain
Source: bioRxiv. 2025 Aug 28:2025.08.22.671845. Preprint. [Version 1] doi: 10.1101/2025.08.22.671845 (PMC12407747; doi:10.1101/2025.08.22.671845)
Supplement: Supplement 3 — Suppl. Fig. 3: Lipid accumulation-induced changes in senescence and inflammation-related genes in astrocytes during aging. A-C) Density plots depicting the gene set enrichment scores for core senescence genes, SASP- and neuroinflammation-related genes, respectively in Plin2 positive and negative astrocytes from the aged brains. [file media-3.pptx]

## Slide 1
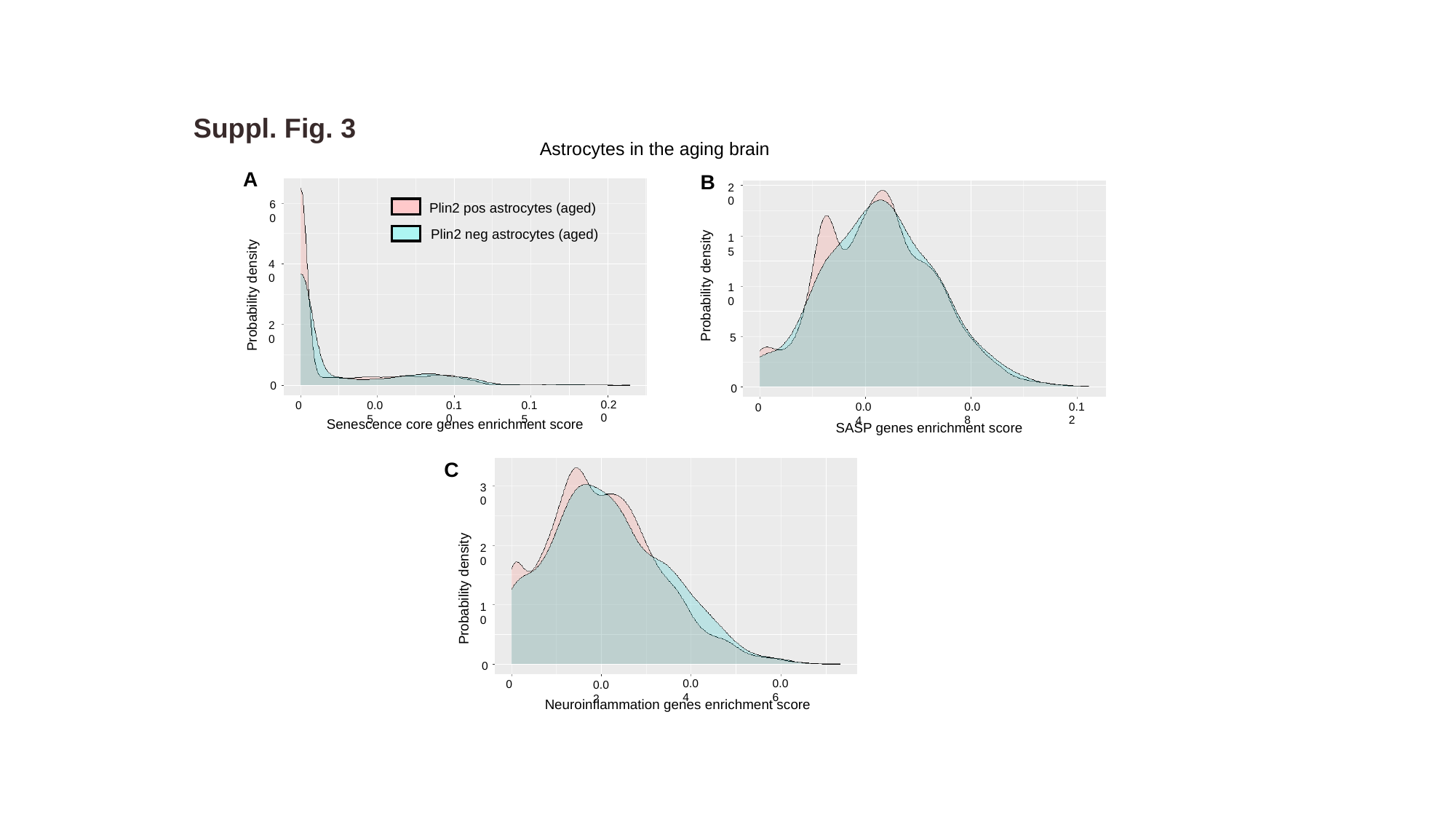

Suppl. Fig. 3
Astrocytes in the aging brain
A
B
60
40
Probability density
20
0
0.20
0.10
0.15
0
0.05
Senescence core genes enrichment score
20
15
Probability density
10
5
0
0.08
0.12
0.04
0
SASP genes enrichment score
Plin2 pos astrocytes (aged)
Plin2 neg astrocytes (aged)
C
30
20
Probability density
10
0
0.06
0.04
0
0.02
Neuroinflammation genes enrichment score
